# *Clostridium sordellii* Lethal-Toxin Autoprocessing and Membrane Localization Activities Drive GTPase Glucosylation Profiles in Endothelial Cells

**DOI:** 10.1128/mSphere.00012-15

**Published:** 2015-11-18

**Authors:** Ryan Craven, D. Borden Lacy

**Affiliations:** a Department of Pathology, Microbiology, and Immunology, Vanderbilt University School of Medicine, Nashville, Tennessee, USA; b The Veterans Affairs Tennessee Valley Healthcare System, Nashville, Tennessee, USA; University of Kentucky

**Keywords:** cytotoxins, membranes, glucosylation, GTPases

## Abstract

*Clostridium sordellii* infections cause gangrene and edema in humans and gastrointestinal infections in livestock. One of the principle virulence factors is TcsL, a large protein toxin which glucosylates host GTPases to cause cytopathic and cytotoxic effects. TcsL has two enzymatic domains, an N-terminal glucosyltransferase domain (GTD) and an autoprocessing domain responsible for release of the GTD within the cell. The GTD can then use its N-terminal membrane localization domain (MLD) for orientation on membranes and modification of GTPases. This study describes the use of conditionally immortalized murine pulmonary microvascular endothelial cells as a model for the study of TcsL functional activities. Point mutations that disrupt the glucosyltransferase, autoprocessing, or membrane localization activities were introduced into a recombinant version of TcsL, and the activities of these mutants were compared to those of wild-type toxin. We observed that all mutants are defective or impaired in cytotoxicity but differ in their modification of Rac1 and Ras. The data suggest a model where differences in GTPase localization dictate cellular responses to intoxication and highlight the importance of autoprocessing in the function of TcsL.

**IMPORTANCE** *Clostridium sordellii* is a bacterium that can infect humans and cause serious disease and death. The principle virulence factor associated with clinical symptoms is a large protein toxin known as lethal toxin. The mechanism of lethal-toxin intoxication is assumed to be similar to that of the homologous toxins from *C. difficile*, but very few studies have been done in the context of endothelial cells, a relevant target in *C. sordellii* infections. This study was designed to test the role of the lethal-toxin enzymatic activities and membrane localization in endothelial cell toxicity and host substrate modification.

## Abstract (precis)

*Clostridium sordellii* is a bacterium that can infect humans and cause serious disease and death. The principle virulence factor associated with clinical symptoms is a large protein toxin known as lethal toxin. The mechanism of lethal-toxin intoxication is assumed to be similar to that of the homologous toxins from *C. difficile*, but very few studies have been done in the context of endothelial cells, a relevant target in *C. sordellii* infections. This study was designed to test the role of the lethal-toxin enzymatic activities and membrane localization in endothelial cell toxicity and host substrate modification.

## INTRODUCTION

*Clostridium sordellii* is a Gram-positive, spore-forming anaerobic bacterium that causes infections in humans and livestock (1). In humans, the bacteria enter at sites of soft tissue trauma and cause infections that lead to gas gangrene, sepsis, and, in up to 70% of cases, death (2–5). *C. sordellii* produces two major toxins, the hemorrhagic toxin (TcsH) and the lethal toxin (TcsL), which are considered the major virulence factors in disease (6, 7). First, animals that are injected with purified toxins develop symptoms that mimic the symptoms of *C. sordellii* infection (6, 8). Second, antibodies that target TcsL and TcsH can protect against tissue damage (9, 10). As this organism causes a toxin-mediated disease, there is therefore interest in understanding the molecular mechanism of toxin action, especially that of TcsL, since not all clinical isolates contain TcsH (11).

While much of what we understand about the TcsH/TcsL mechanism comes from analogy to the homologous TcdA and TcdB toxins from *Clostridium difficile*, there are several reports validating the functional similarities. Toxin activity relies upon binding to a receptor(s) on the cell surface and clathrin-mediated endocytosis into the host cell (12). Maturation of the endosome causes a conformational change in the pore-forming domain of TcsL, causing it to form a pore in the endosomal membrane (13, 14). The autoprocessing domain is activated by host inositol hexakisphosphate and cleaves the glucosyltransferase domain (GTD) (15–18), presumably to permit access to substrates residing at the plasma membrane. The GTD glucosylates small GTPases, predominately Rac, Ras, Ral, and Rap (19–21). The glucosylation leads to cytoskeletal rearrangement and rounding of the cells and also causes the induction of apoptosis (22–25).

Previous studies investigated the different roles of host GTPases in the cellular response to intoxication and showed that glucosylation of H, K, and NRas leads to the apoptosis seen in cells by causing cell cycle arrest (26–28). Studies have also shown that Rac1 glucosylation drives a change in cell morphology through actin cytoskeletal rearrangement, which exerts the cytopathic effects (29). While work has been done to understand the relationship between specific GTPase glucosylation and the cellular effects induced by TcsL, an in-depth look at the role of the toxin functional activities in the context of cellular intoxication has not been reported. We introduced mutations into TcsL to inhibit the glucosyltransferase and autoprocessing enzymatic functions and studied the changes these mutations exhibit in lung endothelial cells.

## RESULTS

### TcsL induces cytotoxicity in mPMVECs.

**FIG 1:**
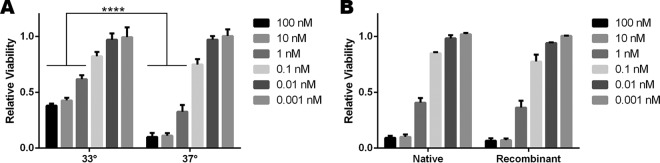
mPMVECs as a model to study cytotoxicity. (A) Murine pulmonary microvascular endothelial cells (mPMVECs) were incubated at 33°C or 37°C and treated with TcsL over a range of concentrations. Cell viability was determined by CellTiter-Glo luciferase 24 h after intoxication. (B) The dose responses for native and recombinant TcsL were identical. Toxins were incubated with mPMVECs for 24 h at 37°C. Relative viability was calculated dividing the signal from intoxicated cells by the signal from those that were mock treated and averaging across three biological replicates. Error bars represent standard errors of the means. ****, *P* < 0.0001.

TcsL cytotoxicity is driven by glucosylation of Ras, and many Ras-related pathways are mutated in standard cell lines to induce immortality. Much of the work done to study the impact of TcsL has used either transformed cell lines or cell lines that do not accurately represent the tissue specificity shown during *C. sordellii* infections. *C. sordellii* infection leads to edema and hypotension, and TcsL induces vascular permeability in the lungs of mice (8). This suggests that lung endothelial cells are a physiologically relevant model for studies of TcsL function. We chose conditionally immortalized murine pulmonary microvascular endothelial cells (mPMVECs), which behave similarly to primary cells when grown at 37°C but are permissive for expression of the simian virus 40 (SV40) large T antigen at 33°C (30). We used a recombinant system to allow expression and purification of TcsL with specific point mutations (12, 18). Cells were treated with TcsL across a range of concentrations at both the permissive temperature (33°C) and the nonpermissive temperature (37°C). TcsL induced significantly higher levels of cytotoxicity at 37°C (Fig. 1A). A comparison between recombinant TcsL and TcsL purified from *C. sordellii* indicates that both forms of TcsL induce similar effects on endothelial cells, with no statistical difference in cytotoxicity levels (Fig. 1B).

### TcsL autoprocessing and glucosyltransferase activities are important for cytotoxicity.

**FIG 2:**
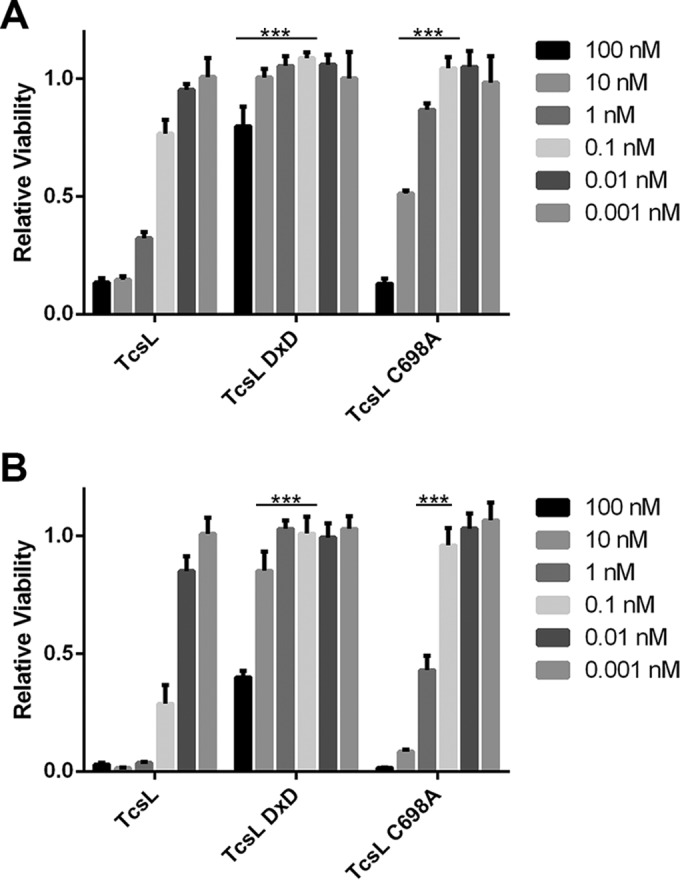
TcsL autoprocessing and glucosyltransferase mutants are impaired in cytotoxicity. Endothelial cells were treated with TcsL, glucosyltransferase-deficient TcsL (TcsL DxD), or autoprocessing-deficient TcsL (TcsL C698A) over a range of concentrations. Cell viability was determined with Glo luciferase 24 h (A) and 48 h (B) after intoxication. Relative viability was calculated by dividing the signal from intoxicated cells by the signal from those that were mock treated and averaging across three biological replicates. Error bars represent standard errors of the means. ***, *P* < 0.001.

To determine the importance of the enzymatic activities of TcsL, mutations were introduced into the active sites of the GTD and the autoprocessing domain. The glucosyltransferase activity was ablated by the introduction of the mutations D286N and D288N (DxD), and the autoprocessing activity was eliminated by introducing a C698A mutation (18, 31, 32) (see Table S1 in the supplemental material). The endothelial cells were treated with TcsL as well as the glucosyltransferase and autoprocessing mutants (DxD and C698A, respectively). Cell viability was determined at 24 and 48 h postintoxication using CellTiter-Glo. When cells were treated with glucosyltransferase-deficient TcsL, no cell death was seen except at the highest concentration tested (Fig. 2). The autoprocessing mutant was attenuated but still induced cytotoxicity. These findings suggest that glucosyltransferase activity is required for cytotoxicity, while the autoprocessing activity is important but not required for induction of cell death.

10.1128/mSphere.00012-15.1 Text S1  Supplemental methods. Details of *in vitro* glucosyltransferase assay and liposome binding assay. Download Text S1, DOCX file, 0.1 MB. Copyright © 2015 Craven and Lacy. 2015 Craven and Lacy This content is distributed under the terms of the Creative Commons Attribution 4.0 International license.

10.1128/mSphere.00012-15.2 Table S1  Plasmid information and primer sequences for TcsL and TcsL mutations. Download Table S1, DOCX file, 0.1 MB. Copyright © 2015 Craven and Lacy. 2015 Craven and Lacy This content is distributed under the terms of the Creative Commons Attribution 4.0 International license.

**FIG 3:**
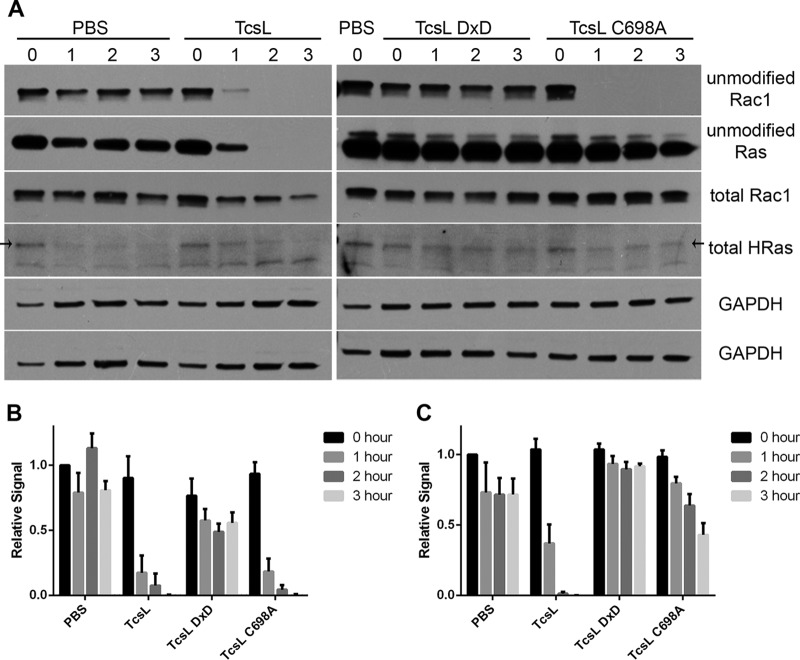
Kinetics of Rac1 versus Ras glucosylation differ when cells are treated with a TcsL autoprocessing mutant. Endothelial cells were treated with 10 nM TcsL, TcsL DxD, TcsL C698A, or phosphate-buffered saline (PBS) only. The cells were incubated for 0, 1, 2, or 3 h before lysis. (A) Lysate supernatants were analyzed by Western blotting using antibodies for unglucosylated Rac and Ras, total Rac, total HRas, and GAPDH. (B and C) Quantification was performed for Rac (B) and Ras (C) signal relative to PBS-treated sample at 0 h. Error bars show standard errors of the means from four replicates.

We next assayed the impact of enzyme mutation on the glucosylation of Rac1 and Ras GTPases in cells. We analyzed endothelial cell lysates that had been treated with 10 nM TcsL, TcsL DxD, and TcsL C698A. Cell lysates were analyzed by Western blots using Rac1 and Ras antibodies that are specific to unglucosylated GTPase (21, 27, 33). Once Rac1 and Ras are modified by TcsL, the antibody can no longer recognize its epitope, and the signal is lost. The cells that were treated with TcsL showed modification after 1 h, and glucosylation of both GTPases was nearly complete by 2 h (Fig. 3). When TcsL DxD was used to intoxicate cells, the loss of the glucosyltransferase activity rendered the mutant unable to glucosylate both Rac1 and Ras. Interestingly, TcsL C698A was able to quickly glucosylate Rac1, similar to wild-type TcsL, but was attenuated in its ability to glucosylate Ras GTPases. The introduction of the autoprocessing mutation did not impact the glucosylation of Rac1 or H-Ras in an *in vitro* assay (see Fig. S1 in the supplemental material). The difference in cellular Rac1 and Ras glucosylation when the autoprocessing activity was ablated suggests that there is a localization difference for Rac1 and Ras.

10.1128/mSphere.00012-15.3 Figure S1  Autoprocessing mutation does not alter glucosylation. Rac1 and HRas were glucosylated using TcsL and TcsL C698A in the presence of UDP-[^14^C]glucose. Download Figure S1, TIF file, 1.9 MB. Copyright © 2015 Craven and Lacy. 2015 Craven and Lacy This content is distributed under the terms of the Creative Commons Attribution 4.0 International license.

### Mutations in the GTD membrane localization domain inhibit TcsL cytotoxicity.

**FIG 4:**
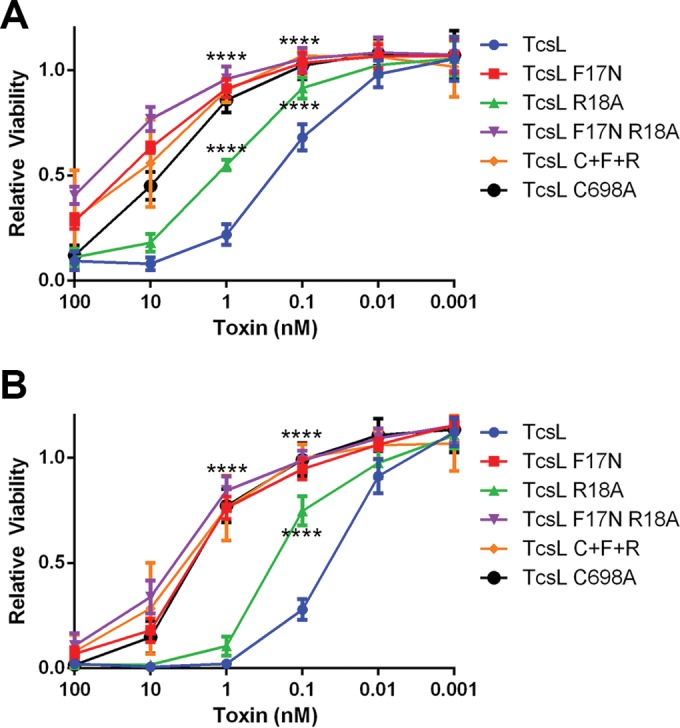
TcsL membrane localization domain is important for cytotoxicity. Endothelial cells were treated with various concentrations of TcsL, TcsL membrane localization mutants (TcsL F17N, TcsL R18A, and TcsL F17N R18A), TcsL C698A, or the triple mutant TcsL F17N R18A C698A (C + F + R). Cell viability was determined with Glo luciferase 24 h (A) and 48 h (B) after intoxication. Relative viability was calculated by comparing the signal to cells that were mock treated and represents the average of three replicates. Error bars represent standard errors of the means. ****, *P* < 0.0001.

Previous reports identified a membrane localization domain (MLD) on the GTD that is involved in the localization of the GTD to the plasma membrane (34, 35). To test the importance of the MLD in TcsL cytotoxicity, F17N and R18A mutations (analogous to mutations made in the study reported in reference 35) were created, as well as a double mutation (F17N R18A). These residues are found on the surface of the MLD and were previously implicated in membrane localization (35), and the double mutant (F17N R18A) shows a defect in membrane association in a liposome binding assay (see Fig. S2 in the supplemental material). A triple mutation (F17N R18A C698A) was also created to determine the impact of both autoprocessing and MLD mutations on cytotoxicity. Endothelial cells were treated with TcsL and TcsL MLD mutants, and cytotoxicity was assessed. Relative to treatments with wild-type TcsL, cytotoxicity was significantly attenuated when cells were treated with the MLD mutants (Fig. 4). The level of attenuation for TcsL MLD mutants is similar to that of TcsL C698A and suggests that TcsL cytotoxicity is dependent upon GTD localization to the cell membrane.

10.1128/mSphere.00012-15.4 Figure S2  MLD mutants disrupt lipid binding. TcsL and TcsL mutants were combined with liposomes, and after incubation the bound toxin was pelleted. The gel (bottom) shows both supernatant and pellet fractions for each toxin and is representative of three separate experiments. The relative values show that the bound toxin level decreases when membrane localization mutations are introduced. *, *P* < 0.05. Download Figure S2, TIF file, 0.1 MB. Copyright © 2015 Craven and Lacy. 2015 Craven and Lacy This content is distributed under the terms of the Creative Commons Attribution 4.0 International license.

**FIG 5:**
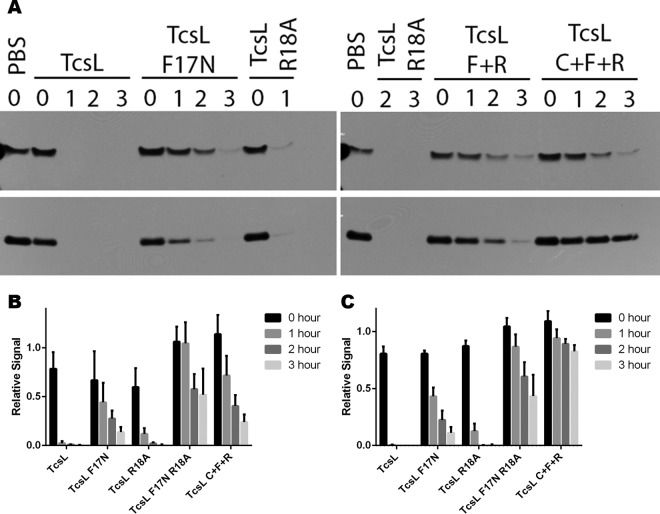
Membrane localization domain is required for efficient Rac modification. Endothelial cells were treated with 10 nM TcsL, TcsL F17N, TcsL R18A, TcsL F17N R18A, or TcsL C698A F17N R18A for 0, 1, 2, or 3 h. Cells were lysed by detergent-free lysis, and supernatants were stained for unmodified Rac and unmodified Ras GTPase (A). The signals relative to untreated samples were quantified for unmodified Rac (B) and unmodified Ras (C) and averaged across three replicates. Error bars represent standard errors of the means.

To understand how MLD mutations affect the mechanism of cellular response, we next assayed the glucosylation of Rac1 and Ras by TcsL MLD mutants. Cell lysates were obtained from endothelial cells treated with TcsL and TcsL MLD mutants and analyzed by Western blotting for Rac1 and Ras glucosylation. While TcsL was able to quickly glucosylate Rac1 and Ras, the MLD mutants were attenuated in their capacity to glucosylate substrates in the cell (Fig. 5). Both TcsL F17N and TcsL R18A were delayed in their glucosylation of Rac1 and Ras, and the double mutant, TcsL F17N R18A, was the most attenuated in its glucosylation of Rac1 and Ras. The triple mutant (TcsL F17N R18A C698A) was also inhibited in both Rac1 and Ras glucosylation. These observations indicate that the MLD interaction with the membrane is important for the glucosylation of both Rac1 and Ras and that the MLD mutation prevents the autoprocessing-deficient TcsL from efficiently glucosylating Rac1.

## DISCUSSION

The purpose of our study was to understand the impact of the different TcsL enzymatic activities in the context of lung endothelial cells. Although many cell lines have been used in the study of TcsL-induced effects, we wanted to find a cell line that would be a good model for the effects seen during infection. Symptoms seen commonly in *C. sordellii* infection are edema, hypotension, and multiorgan failure, indicating that the toxins act strongly upon host microvasculature. Our use of conditionally immortalized murine pulmonary microvascular endothelial cells allowed us to induce a primary cell-like state that removed the potential interference of common mutations associated with immortalization. Figure 1 shows that TcsL is a potent cytotoxin in these cells, which allowed us to assess the impact of enzymatic activities in the cell death mechanism.

When endothelial cells were treated with TcsL and the DxD and C698A mutants, we saw that cytotoxicity was inhibited for the glucosyltransferase mutant and impaired in the autoprocessing mutant. The inhibition of cytotoxicity when treating cells with TcsL DxD supports previous research showing that glucosylation of the host cell GTPases leads to arrest of the cell cycle and induction of apoptosis (28). The decrease in cytotoxicity seen with TcsL C698A suggests that the autoprocessing activity is important in TcsL cytotoxicity (Fig. 2).

Previous studies investigating the role of autoprocessing in the *C. difficile* toxins suggested that preventing GTD release through inactivation of the autoprocessing activity has only modest effects on cytopathic responses (36) and, in the case of TcdB, no impact on toxin-induced necrosis (37). The observations raise the question of why the autoprocessing activity has been retained in this family of toxins. A key difference between TcsL and the *C. difficile* toxins is that TcsL is more active in the modification of Ras GTPases (21, 33), and Ras inactivation has been linked to TcsL-induced cell death (28). We therefore assayed whether TcsL and the TcsL C698A mutant were capable of modifying both Rac and Ras. The experiments in Fig. 3 reveal that while autoprocessing is not required for the modification of Rac, it is important for the efficient modification of Ras.

The differential ability to glucosylate the GTPases when the GTD is not cleaved from the holotoxin suggests a difference in the localization of Rac and Ras GTPases. It has been reported that Rac cycles to the endosomes where it is activated before trafficking back to the membrane (38). TcsL C698A does not release the GTD, and it remains bound to the endosome. The GTD may then encounter Rac that has been trafficked to the endosome for activation. Ras GTPases, however, are trafficked to and found in abundance at the plasma membrane after translation (39). We therefore propose that the GTD that remains tethered to the endosome does not immediately encounter and glucosylate Ras proteins. We propose that the slow Ras glucosylation is due to endosomal membranes, with the tethered GTD, recycling back to the cell surface. The delay in Ras glucosylation is enough to cause the decrease in cytotoxicity.

Previous studies identified an MLD on the TcsL GTD that is conserved across all large clostridial toxins and important in membrane localization. The MLD may be important for GTD to be inserted into the plasma membrane and may help tether it to the membrane (40, 41). We looked at the impact of MLD point mutations on both cytotoxicity (Fig. 4) and the glucosylation of host GTPases (Fig. 5). Our work shows that the introduction of MLD mutations inhibits the ability to induce cytotoxicity and delays the glucosylation of both Rac and Ras GTPases, supporting the importance of the MLD in tethering GTD to the cell membrane, where it can interact with and glucosylate the GTPases. Interestingly, when we combined the MLD mutations with the autoprocessing mutation, we saw a decrease in cytotoxic ability as well as glucosylation of both Rac and Ras. While TcsL C698A was able to efficiently glucosylate Rac, the loss of efficient Rac glucosylation with the introduction of MLD mutations suggests that it is not enough for the GTD to be tethered to the endosome but also relies on the ability of the GTD to interact with the endosomal membrane through the MLD.

Our studies have shown the impact of mutating the enzymatic domains of TcsL. While the glucosyltransferase activity is needed for Rac modification and cytotoxicity, autoprocessing-deficient TcsL is impaired in cytotoxicity but efficient in its modification of Rac. We also show that the interaction with the cell membrane, not just proximity, is needed for efficient glucosylation of GTPases. The increased understanding of each toxin domain during host intoxication provides a foundation for more targeted approaches to study toxin-induced cellular events.

## MATERIALS AND METHODS

### Toxin cloning, expression, and purification.

TcsL was amplified from *C. sordellii* strain JGS6382 and inserted into a BMEG20 vector (MobiTec) using BsrGI/KpnI restriction digestion sites in the vector, as reported previously (18). Point mutations were introduced into the glucosyltransferase domain (F17N, R18A, D286N, and D288N) and autoprocessing domain (C698A) from the wild-type recombinant TcsL sequence using a QuikChange mutagenesis protocol. Construct names and primers used are found in Table S1 in the supplemental material. Recombinant toxins were expressed in *Bacillus megaterium* using 35 ml of overnight culture seeded into 1 liter of LB. Expression of toxin was induced at an optical density at 600 nm (OD_600_) of roughly 0.5 with 5 g d-xylose and grown for 4 h at 37°C with shaking at 220 rpm. The cells were pelleted and resuspended in lysis buffer (20 mM KP_i_ [pH 7], 500 mM NaCl, DNase, and protease inhibitors [Sigma]). The bacteria were passed through an Emulsiflex homogenizer for lysis, and centrifugation was performed at 48,000 × *g* for 30 min. Supernatants were run over Ni affinity anion exchange columns with a gradient from 20 mM Tris (pH 8) to 20 mM Tris (pH 8)–600 mM NaCl and on Superdex 200 size exclusion columns, and toxins were eluted in 20 mM HEPES (pH 7)–50 mM NaCl.

Native TcsL was purified from *C. sordellii* strain JGS6382, obtained from David Aronoff (Vanderbilt University). Expression and purification were done as previously described (37).

### Cell culture.

Conditionally immortalized murine pulmonary microvascular endothelial cells (mPMVECs) were obtained from the laboratory of Ambra Pozzi (Vanderbilt University). The cells were grown at 33°C using EGM-2 (Lonza) supplemented with 10 ng/ml gamma interferon (IFN-γ) in 5% CO_2_. For use, mPMVECs were transferred to 37°C for overnight growth in EGM-2 medium without IFN-γ.

### Viability assays.

mPMVECs were plated at 2,500 cells/well in black-wall 96-well plates. The cells were treated with toxin the following day and incubated at 37°C in 5% CO_2_ for either 24 or 48 h. After incubation with toxin, viability was assessed by measuring the amount of ATP present by addition of CellTiter-Glo (Promega), and the luminescence was read using a BioTek Synergy 4 plate reader. Relative viability was calculated by setting the readings for mock-treated samples as 100%.

### Western blot analysis.

Cell lysates were prepared from mPMVECs that had been plated at 200,000 cells/ml in 10-cm dishes the day before. Cells were intoxicated with 10 nM toxin and incubated at 37°C for 0, 1, 2, and 3 h before manual lifting from the dish. Cells were washed and resuspended in lysis buffer (250 mM sucrose, 10 mM Tris [pH 7.4], 3 mM imidazole) and passed through a 27-gauge needle 25 times. The lysates were clarified by centrifugation. Samples were run on SDS-PAGE gels (Bio-Rad) and transferred to polyvinylidene difluoride (PVDF) for Western analysis. Blots were probed with antibodies for unmodified Rac1 (610651; BD), total Rac1 (clone 23A8; Millipore), unmodified Ras (ab52939; Abcam), total HRas (sc-520; Santa Cruz), and GAPDH (sc-25778; Santa Cruz). Horseradish peroxidase (HRP)-conjugated anti-mouse and anti-rabbit antibodies (7076 and 7074, respectively; Cell Signaling) were applied as secondary antibodies, and the blots were visualized using Pierce enhanced chemiluminescence Western blotting substrate (Thermo) and exposure to film. Film was scanned using the Odyssey Licor imaging system and analyzed using Image Studio Lite.

### Statistics.

Statistics were performed using two-way analysis of variance (ANOVA), and *P* values were determined using Dunnett’s multiple comparisons test on GraphPad Prism.
