# Supplementary material for: Clostridium sordellii Lethal-Toxin Autoprocessing and Membrane Localization Activities Drive GTPase Glucosylation Profiles in Endothelial Cells
Source: mSphere. 2015 Nov 18;1(1):e00012-15. doi: 10.1128/mSphere.00012-15 (PMC4863631; doi:10.1128/mSphere.00012-15)
Supplement: Text S1 [file sph001160034s1.docx]

**Supplemental information**

**Title:**

*Clostridium sordellii* Lethal Toxin Autoprocessing and Membrane Localization Activities Drive GTPase Glucosylation Profiles in Endothelial Cells

**Authors:**

Ryan Craven^a^, D. Borden Lacy^a,b,^#

**Methods**

*In vitro* glucosyltransferase assay:

TcsL and TcsL C698A were incubated in buffer containing 50 mM HEPES, 100 mM KCl, 1 mM MnCl_2_, 2 mM MgCl_2_, 0.1 mg/mL BSA (pH 7.5) with 24 uM UDP-[^14^C]glucose (250 mCi/mmol, PerkinElmer) and 2 uM GST-tagged Rac1 or HRas for 1 hour at 37° C. Reactions were stopped by adding loading buffer and boiling. Samples were separated by SDS-PAGE, and the dried gels were imaged using a Typhoon FLA 7000 phosphoimaging scanner.

Liposome binding assay:

Liposomes were prepared using 30% DOPE, 20% cholesterol, 20% egg PC, and 30% brain PS (Avanti Polar Lipids) by combining lipid solutions in chloroform and dried by nitrogen gas and vacuum. The dried lipids were resuspended to 10 mM final concentration in 20 mM HEPES, pH 7.5, 100 mM KCl and freeze-thawed. The lipids were then passed through an extruder using a 0.2 uM filter repeatedly. Toxin was cleaved using 2 uM toxin, 50 mM DTT, and 5 mM IP6 in 20 mM HEPES pH 7 buffer and incubated at 37° C for 2 hours. The cleaved toxin was dialyzed against 20 mM HEPES, pH 7.5, 100 mM KCl to remove IP6 and DTT. Liposome binding reactions were prepared using 3 mM liposomes and 0.5 uM dialyzed toxin incubated in 20 mM HEPES, pH 7.5, 100 mM KCL buffer containing 1 mM MgCl_2_ and 1 mM CaCl_2_ for 1 hour at 37° C. The reactions were separated by centrifugation at 436,000 g for 1 hour at 23° C. Pellets were resuspended in the same volume of buffer with 1% SDS added. Samples were separated by SDS-PAGE and stained by SimplyBlue SafeStain Coomassie (ThermoFisher).
