# Supplementary material for: Clostridium sordellii Lethal-Toxin Autoprocessing and Membrane Localization Activities Drive GTPase Glucosylation Profiles in Endothelial Cells
Source: mSphere. 2015 Nov 18;1(1):e00012-15. doi: 10.1128/mSphere.00012-15 (PMC4863631; doi:10.1128/mSphere.00012-15)
Supplement: Table S1 [file sph001160034st2.docx]

**Table S1**

| **Plasmid Name** | **Protein Expressed** | **Primer Sequences Used** |
| --- | --- | --- |
| pBL552 | TcsL in BMEG20 vector |  |
|  |  |  |
| pBL685 | TcsL C698A | 5'-GTCTATAGAAATAAATTTACTAGGAGCTAATATGTTCAGCTATAACGTTAATG-3' |
|  |  | 5'-CATTAACGTTATAGCTGAACATATTAGCTCCTAGTAAATTTATTTCTATAGAC-3' |
| pBL686 | TcsL D286N, D288N | 5'-GGTGGGGTATATTTAAATGTTAATATGTTACCAGGTATAC-3'  5'-GTATACCTGGTAACATATTAACATTTAAATATACCCCACC-3' |
| pBL691 | TcsL R18A | 5'-GGCATATGTAAAATTTGCTATTCAAGAAGATGAGTACGTAGC-3'  5'-GCTACGTACTCATCTTCTTGAATAGCAAATTTTACATATGCC-3' |
| pBL692 | TcsL F17N | 5'-GGCATATGTAAAAAATCGTATTCAAGAAGATGAGTACGTAGC-3' |
|  |  | 5'-GCTACGTACTCATCTTCTTGAATACGATTTTTTACATATGCC-3' |
| pBL745 | TcsL F17N, R18A | 5'-GGCATATGTAAAAAATGCTATTCAAGAAGATGAGTACGTAGC-3' |
|  |  | 5'-GCTACGTACTCATCTTCTTGAATAGCATTTTTTACATATGCC-3' |
| pBL746 | TcsL F17N, R18A, C698A | sequences above for pBL745 and pBL685 |
|  |  |  |

**Table 1:** **Plasmid information and primer sequences for TcsL and TcsL mutations**
