# Supplementary figures and images for: Clostridium sordellii Lethal-Toxin Autoprocessing and Membrane Localization Activities Drive GTPase Glucosylation Profiles in Endothelial Cells
Source: mSphere. 2015 Nov 18;1(1):e00012-15. doi: 10.1128/mSphere.00012-15 (PMC4863631; doi:10.1128/mSphere.00012-15)

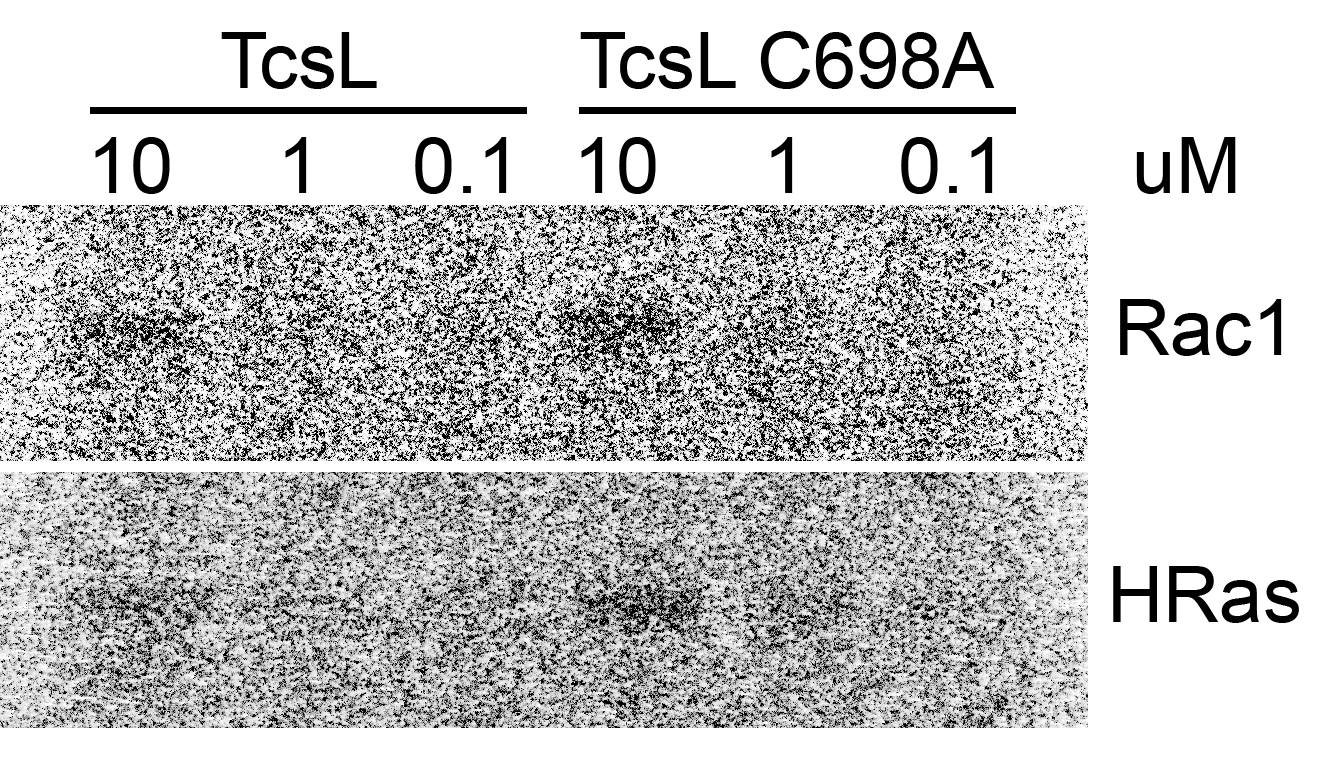

Supplement: Figure S1 [file sph001160034sf3.tif]

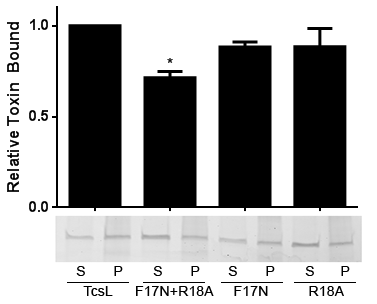

Supplement: Figure S2 [file sph001160034sf4.tif]
